# Effect of Tongxinluo on Podocyte Apoptosis via Inhibition of Oxidative Stress and P38 Pathway in Diabetic Rats

**DOI:** 10.1155/2016/5957423

**Published:** 2016-09-08

**Authors:** Fangqiang Cui, Yanbin Gao, Wenjing Zhao, Dawei Zou, Zhiyao Zhu, Xiaoming Wu, Nianxiu Tian, Xiaolei Wang, Jing Liu, Yu Tong

**Affiliations:** ^1^School of Traditional Chinese Medicine, Capital Medical University, No. 10, Youanmenwai, Xitoutiao, Fengtai District, Beijing 100069, China; ^2^Beijing Key Lab of TCM Collateral Disease Theory Research, No. 10, Youanmenwai, Xitoutiao, Fengtai District, Beijing 100069, China; ^3^Department of Nephrology, Beijing Traditional Chinese Medicine Hospital, Capital Medical University, 23 Meishuguanhou Street, Dongcheng District, Beijing 100010, China

## Abstract

Diabetic nephropathy (DN) has been the leading cause of end-stage renal disease (ESRD). Podocyte apoptosis is a main mechanism of progression of DN. It has been demonstrated that activated P38 and caspase-3 induced by oxidative stress mainly account for increased podocyte apoptosis and proteinuria in DN. Meanwhile, Tongxinluo (TXL) can ameliorate renal structure disruption and dysfunction in DN patients in our clinical practice. However, the effect of TXL on podocyte apoptosis and P38 pathway remains unclear. To explore the effect of TXL on podocyte apoptosis and its molecular mechanism in DN, our in vivo and in vitro studies were performed. TXL attenuated oxidative stress in podocyte in DN in our in vivo and in vitro studies. Moreover, TXL inhibited the activation of P38 and caspase-3. Bcl-2 and Bax expression was partially restored by TXL treatment in our in vivo and in vitro studies. More importantly, TXL decreased podocyte apoptosis in diabetic rats and high glucose cultured podocyte. In conclusion, TXL protects podocyte from apoptosis in DN, partially through its antioxidant effect and inhibiting of the activation of P38 and caspase-3.

## 1. Introduction

Diabetic nephropathy (DN), as a common and serious microvascular complication of diabetes mellitus (DM), has been the leading cause of end-stage renal disease (ESRD) [1]. Podocyte injury plays a key role in increased microalbuminuria in DN. Previous studies have demonstrated that the number of podocytes is significantly decreased in DN [2–4] and podocyte apoptosis is the main mechanism of podocyte loss [5].

Evidence suggests that reactive oxygen species (ROS) production is significantly increased in the kidney of DM and oxidative stress plays a critical role in the initiation and progression of DN [6–8]. Meanwhile, previous studies have demonstrated that ROS was increased in podocyte [9] and increased ROS production promoted podocyte apoptosis in DN [10]. Moreover, ROS can activate P38 MAPK pathway, which may be an important pathological mechanism of podocyte apoptosis induced by oxidative stress [5, 11, 12]. Thus, the inhibition of the activation of P38 pathway and ROS-induced podocyte apoptosis may be a potential targeted therapy for DN in the future.

TXL, a new kind of Chinese herbal compound, has been widely used in clinical practice in China in recent years. It has been demonstrated that TXL has beneficial effect for cardiovascular diseases and cerebrovascular diseases [13, 14]. More importantly, TXL exhibits its antioxidant properties in the treatment of cardiovascular diseases and cerebrovascular diseases in previous studies [15, 16]. Moreover, ginsenoside Rg1, a major active component of TXL, has therapeutic effect against DM-induced myocardial damage through its antioxidant effect [17]. Meanwhile, TXL can significantly decrease proteinuria and ameliorate renal dysfunction in DN patients in our clinical practice. However, the effect of TXL on oxidative stress in podocyte and ROS-induced podocyte apoptosis in DN remains elusive.

## 2. Materials and Methods

### 2.1. Preparation of TXL

TXL superfine powder was provided by Shijiazhuang Yiling pharmaceutical Co. (Hebei China). The security of TXL has been approved by the State Food and Drug Administration in China. The species, origin, harvest time, medicinal parts, and concoction methods of each component in TXL have been strictly standardized. Moreover, the herbal drugs of TXL were seriously authenticated and standardized through their marker compounds according to the Chinese Pharmacopoeia (2005). All components of TXL were ground to superfine powder with a diameter ≤ 10 *μ*m. In our in vivo study, TXL superfine powder was dissolved in aqua distillate and administrated for DN rats at 60 mg/kg each day. In our in vitro study, TXL superfine powder was dissolved in DMEM/low glucose medium and filtrated by a 0.22 *μ*m micropore filter. The supernatant was collected and then was used for our in vitro study.

### 2.2. Animals

Our in vivo experiment was performed in accordance with the National Institutes of Health Guide for the Care and Use of Laboratory Animals and approved by the Institutional Animal Care and Use Committee at Capital Medical University. Male Sprague-Dawley rats, aged 8 weeks and weighting 180–200 g, were purchased from Chinese Academy of Medical Sciences (Beijing, China). All rats were housed in a conventional environment with a regular 12 h light/dark cycle and 24 ± 1°C temperature. A total of 36 rats were randomly divided into three groups: normal control group (NC group *n* = 12), diabetic nephropathy group (DN group *n* = 12), and DN with TXL group (TXL group *n* = 12). The rats of DN group and TXL group were intraperitoneally injected with STZ (Streptozocin) at 60 mg/kg and their tail vein blood was collected. When their serum glucose ≥ 16.7 mmoL/L, the diabetic rats model was considered to be successful. The rats of NC group were injected with equal volume of vehicle (0.1 M citrate buffer, pH 4.5) as normal control. Then the treatment of different groups was immediately started after diabetic rats were established and lasted for 12 weeks. The rats of TXL group were treated with TXL solution (TXL superfine powder, 0.75 g·kg^−1^·d^−1^, gavage) and the rats of NC group and DN group were treated with equal volume of vehicle (normal saline, NS, gavage). All rats were provided with free access to food and water throughout the experiment. At the end of 12 weeks after STZ, all rats were sacrificed and renal cortex was collected. Three renal cortexes of 12 rats in each group were randomly selected for purposed experiments in our in vivo study.

### 2.3. Cell Culture

The conditionally immortalized mouse podocyte line was provided by National Platform of Experimental Cell Resources for Sci-Tech. To induce podocyte proliferation, podocytes were cultured in DMEM/low glucose (Hyclone, Logan, UT, United States) medium containing 10% fetal bovine serum (Excel Biology, Shanghai, China) and recombinant IFN-*γ* (PEPROTECH, London, UK) at 33°C. Then podocytes were cultured in DMEM/low glucose medium without IFN-*γ* at 37°C for cell differentiation. When they grew to about 80% confluence, podocytes were maintained in serum-free conditions for 24 h and then used in our in vitro study. To detect the optimum concentration of TXL on podocyte apoptosis, podocytes were treated with different concentrations of TXL (10, 25, 50, 100, 150, and 200 *μ*g/mL) for 24 h and then were used for MTT assay and Hoechst 33258 staining in our study. In our in vitro study, the podocytes were divided into three groups: normal control group (NC group), high glucose group (HG group), and HG with Tongxinluo group (TXL group). Podocytes of NC group were treated with DMEM medium containing 5.5 mmol/L glucose + 24.5 mmol/L mannitol. Podocytes of HG group were treated with DMEM medium containing 5.5 mmol/L glucose + 24.5 mmol/L glucose, while cells in TXL group were cultured in DMEM medium containing 30 mmol/L glucose + 100 *μ*g/mL TXL. All experimental groups were treated for 24 h and podocytes were collected and used for purposed experiments.

### 2.4. MTT Assay

Cells were cultured in 96-well plate. When grown at 5000 cells/well, podocytes were treated with different concentrations of TXL (10, 25, 50, 100, 150, and 200 *μ*g/mL) for 24 h. MTT solution at 0.5 mg/mL was added to 96-well plate. After being incubated with MTT solution for 4 h, podocytes were treated with DMSO to dissolve the purple crystals of Formosan. The optical density (OD) was detected by a spectrophotometer at 570 nm. The OD of podocyte in different groups was collected and used for statistical analysis. MTT assay of each group was performed at least in triplicate.

### 2.5. Determination of ROS

The ROS production in podocyte was detected by Reactive Oxygen Species Assay Kit (Biyotime Company, Jiangsu, China). Podocytes were cultured on 24-well plate and treated with different cultured medium for 24 h. Then podocytes were washed twice with cold PBS and treated with probe 2′,7′-dichlorofluorescein diacetate (DCFH-DA) for 30 min at room temperature. Cells were observed under fluorescent microscope. The fluorescence intensity was recorded and used for statistical analysis.

### 2.6. Western Blot

Renal cortex and cultured podocyte were lysed by lysis buffer on ice. Equal amounts of protein (20 *μ*g per lane) were subjected to SDS-PAGE and then transferred to polyvinylidene difluoride membranes. The membranes were blocked by 5% nonfat dry milk in PBS + 0,05% Tween 20. After that, the membranes were incubated with primary antibodies at 4°C overnight. After washing with PBS, peroxidase secondary antibody was added and incubated for 1 h at room temperature. Antibodies are the following: anti-Nox4 antibody (Abcam, UK), anti-P38 antibody (Abcam, UK), anti-P-P38 antibody (Abcam, UK), anti-Bax antibody (Abcam, UK), anti-Bcl-2 antibody (Abcam, UK), and anti-cleaved caspase-3 antibody (Abcam, UK). The blots were visualized with LumiGLO reagent and peroxide, followed by exposure to X-ray film. Western Blot analyses were performed at least in triplicate.

### 2.7. Real-Time RT-PCR

Total RNA of renal cortex and cultured podocyte was isolated using the TRIzol reagent (Invitrogen) according to the manufacturer's instructions. The RNA was reverse-transcribed into cDNAs by the SuperScript RT kit (Invitrogen). RT-PCR was performed using SYBR green real-time quantitative reverse transcription PCR (qRT-PCR) (Applied Biosystems) and the relative mRNA levels were calculated by the 2^−ΔΔCt^ method. The sequences of primers are the following: mice Nox-4: forward prime, CAGAGACATCCAATCATTCCAGTG, reverse prime, CTGGATGTTCACAAAGTCAGGTCT; mice Bax: forward prime, CAGGGTTTCATCCAGGATCGAGCAGG, reverse prime, CGGGGGGAGTCCGTGTCCACGTCAG; mice Bcl-2: forward prime: CCAGCGTGTGTGTGCAAGTGTAAAT, reverse prime, ATGTCAATCCGTAGGAATCCCAACC; rat Nox-4: forward prime, CATTGGGGTCACTCCGTTTG, reverse prime, CCACTGGAATGATTGGATGTCTC; rat Bax: forward prime, GGTGGTTGCCCTTTTCTACTTTGC, reverse prime, GCTCCCGGAGGAAGTCCAGTG; rat Bcl-2: forward prime, GGGCTACGAGTGGGATACTGGAG, reverse prime, CGGGCGTTCGGTTGCTCT.

### 2.8. Immunofluorescence

Cells of different groups were cultured on glass slides in 24-well plates. Cells were fixed with 4% paraformaldehyde for 30 min and then were blocked by 2.5% dunk serum. After washing with PBS, podocytes were incubated with primary antibodies at 4°C overnight. After that, secondary antibodies were added in cells at room temperature for 2 h, followed by counterstaining with DAPI. Cells were observed under fluorescent microscope.

### 2.9. TUNEL

The apoptotic cells of glomerulus were detected by TUNEL detection kit (Roche Diagnostics, Mannheim, Germany) (Nanjing Jiancheng Bioengineering Institute, Nanjing, China) according to the manufacturer's instruction. Renal tissue sections (4 *μ*m) were incubated with protease K for 30 min at 37°C and then washed by 3% H_2_O_2_ for 10 min at room temperature. The mixture of terminal deoxynucleotidyl transferase (TdT) and biotin-dUTP was added to the renal sections. After that, the sections were incubated with streptavidin-horseradish peroxidase and then were stained by DAB. The apoptotic cells were observed by fluorescent microscope. Apoptotic cells residing on the outer surface of GBM were considered as podocyte. The total number of apoptotic podocytes in each group was counted by examining 10 glomeruli and then they were used to calculate apoptotic index.

### 2.10. Flow Cytometry Analysis

Flow cytometry was used to detect podocyte apoptosis in our in vitro study. After being treated with different cultured medium for 24 h, podocytes were washed with cold PBS and collected through centrifugation at 2000 rpm for 5 min. The collected cells were then washed twice with PBS and resuspended with binding buffer. After that, podocytes were treated with annexin V-FITC and PI. Apoptotic cells were collected and analyzed through FACS scan. The rate of apoptotic podocyte was calculated and used for statistical analysis. Flow cytometry analyses of different groups were performed at least in triplicate.

### 2.11. Hoechst 33258 Staining

Hoechst 33258 staining was used for detecting podocyte apoptosis in our in vitro study. Cells were cultured on the cover glasses in 24-well plates. When grown to 80% confluence, podocytes were treated with different medium for 24 h. After that, cells were fixed using 4% paraformaldehyde for 30 min. Podocytes were then stained with Hoechst 33258 for 30 min. Apoptotic podocytes were observed by fluorescent microscope. The number of apoptotic cells was calculated in 10 random fields and used for statistical analysis.

## 3. Results

### 3.1. The Cytotoxicity and Optimum Concentration of TXL for Cultured Podocyte

**Figure 1:**
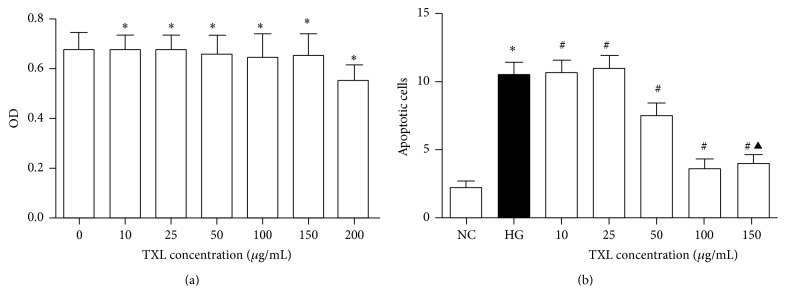
The cytotoxicity and optimum concentration of TXL for cultured podocyte. (a) Comparison of OD detected by MTT assay in cultured podocyte. The result showed that, compared with NC group, the OD had not significantly changed with TXL at the concentrations of 10, 25, 50, 100, and 150 *μ*g/mL (*P* > 0.05); TXL at the concentration of 200 *μ*g/mL significantly decreased the OD of podocyte (*P* < 0.05). (b) Comparison of apoptotic cells detected by Hoechst 33258 staining in cultured podocyte of different groups. The result showed that HG significantly increased the number of apoptotic cells compared with NC group (*P* < 0.05); TXL at the concentration of 10 and 25 *μ*g/mL had no effect on podocyte apoptosis compared with HG group (*P* > 0.05); TXL at the concentration of 50, 100, and 150 *μ*g/mL significantly decreased the number of apoptotic podocytes (*P* < 0.05); the number of apoptotic podocytes had not significantly changed when TXL concentration was added to 150 *μ*g/mL compared with TXL at 100 *μ*g/mL (*P* > 0.05). ^*∗*^ *P* < 0.05 versus NG. ^#^ *P* < 0.05 versus HG. ^▲^ *P* < 0.05 versus TXL (100 *μ*g/mL).

MTT assay was performed to detect TXL cytotoxicity on cultured podocyte in our study. Our results showed that TXL at 10, 25, 50, 100, and 150 *μ*g/mL had no cytotoxicity on podocyte. When the concentration of TXL was added to 200 *μ*g/mL, cell viability was significantly decreased compared with NC group (Figure 1(a)). In order to filter an optimum concentration of TXL on podocyte apoptosis, podocytes were treated with different concentrations of TXL (10, 25, 50, 100, and 150 *μ*g/mL) and podocyte apoptosis was detected by Hoechst 33258 staining. In our study, HG significantly increased podocyte apoptosis compared with NC group. TXL at the concentration of 10 and 25 *μ*g/mL had no effect on podocyte apoptosis compared with HG group. TXL at the concentration of 50, 100, and 150 *μ*g/mL significantly reduced podocyte apoptosis compared with HG group in our in vitro study. More importantly, we found that TXL at 100 *μ*g/mL was the optimum concentration on podocyte apoptosis (Figure 1(b)).

### 3.2. TXL Suppressed Oxidative Stress in Podocyte in Diabetic Rats and High Glucose Cultured Podocyte

**Figure 2:**
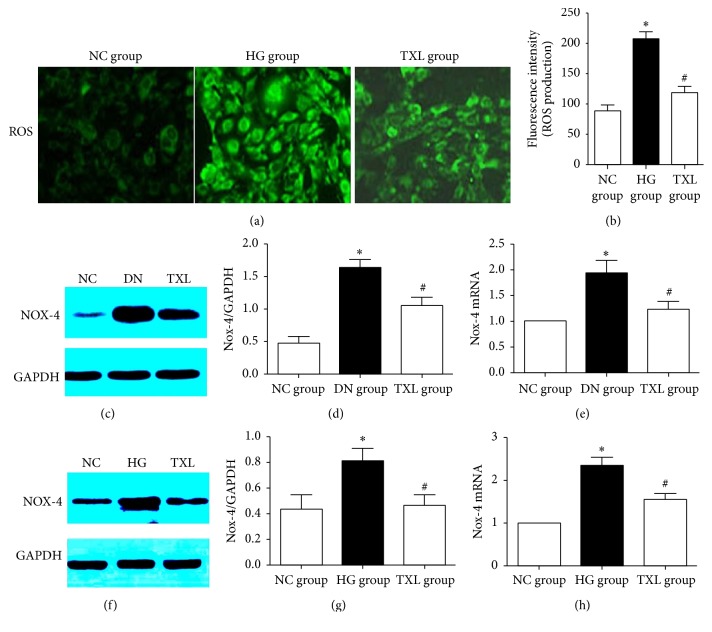
Effect of TXL on oxidative stress in podocyte in vitro and in vivo. (a) Representative photograph of ROS production in podocyte in different groups. (b) Comparison of ROS production in podocyte in different groups. The result showed that ROS production in podocyte of HG group was significantly increased compared with NC group (*P* < 0.05); ROS production in podocyte of TXL group was significantly decreased compared with HG group (*P* < 0.05). (c) Representative band of Nox-4 protein in renal cortex of rats. (d) Comparison of the grey values of Nox-4 protein in renal cortex of rats (*n* = 3). The result showed that the grey value of Nox-4 protein of DN group was significantly increased compared with NC group (*P* < 0.05); the grey value of Nox-4 protein was significantly decreased in TXL group compared with DN group (*P* < 0.05). (e) Comparison of mRNA levels of Nox-4 in renal cortex of rats (*n* = 3). The result showed that Nox-4 mRNA expression was significantly increased in DN group compared with NC group (*P* < 0.05); Nox-4 mRNA expression was significantly decreased in TXL group compared with DN group (*P* < 0.05). (f) Representative band of Nox-4 protein in cultured podocyte. (g) Comparison of the grey values of Nox-4 protein in cultured podocyte (*n* = 3). The result showed that the grey value of Nox-4 protein was significantly increased in HG group compared with NC group (*P* < 0.05); the grey value of Nox-4 protein was significantly decreased in TXL group compared with HG group (h). Comparison of mRNA levels of Nox-4 in cultured podocyte (*n* = 3). The result showed that Nox-4 mRNA expression was significantly increased in HG group compared with NC group (*P* < 0.05); Nox-4 mRNA expression was significantly decreased in TXL group compared with HG group (*P* < 0.05). ^*∗*^ *P* < 0.05 versus NG. ^#^ *P* < 0.05 versus HG.

ROS production in cultured podocyte was detected in our in vitro study. Our results showed that ROS production of HG group was significantly increased compared with NC group. TXL significantly decreased ROS production in high glucose cultured podocyte (Figures 2(a) and 2(b)). Nox-4, a member of NAPDH oxidase family, plays a crucial role in ROS-induced podocyte apoptosis in DN [18, 19]. Herein, Nox-4 expression in renal cortex of diabetic rats and high glucose cultured podocyte was detected in our study. Nox-4 protein expression of diabetic rats and high glucose cultured podocyte, detected by Western Blot, was significantly increased compared with NC group. Similarly, Nox-4 mRNA expression of diabetic rats and high glucose cultured podocyte, detected by RT-PCR, was significantly increased compared with NC group. Moreover, TXL markedly decreased Nox-4 mRNA and protein expression in both renal cortex of diabetic rats and high glucose cultured podocyte (Figures 2(c)–2(h)).

### 3.3. TXL Inhibited the Activation of P38 in Diabetic Rats and in High Glucose Cultured Podocyte

**Figure 3:**
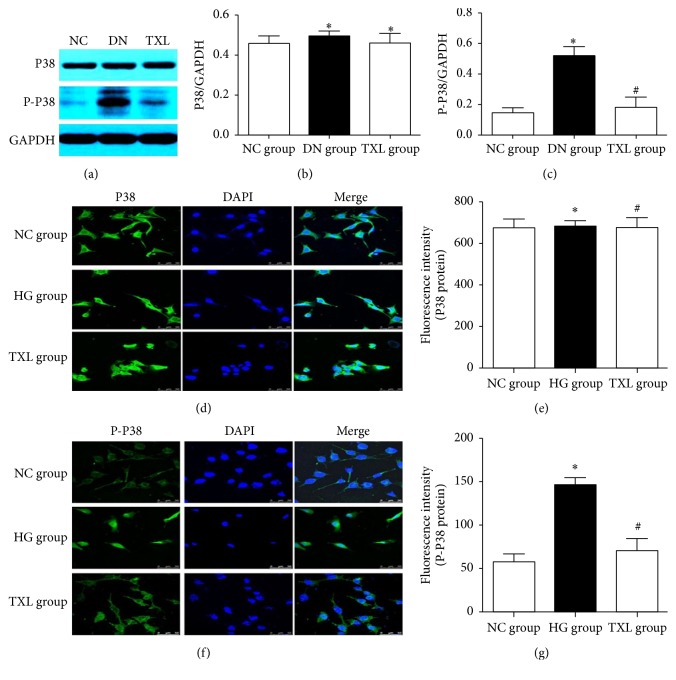
Effect of TXL on P38 activation in podocyte in vivo and in vitro. (a) Representative band of P38 protein and P-P38 protein in renal cortex of rats. (b) Comparison of the grey values of P38 protein in renal cortex of rats (*n* = 3). The result showed that the grey value of P38 protein in DN group and TXL group had not significantly changed compared with NC group (*P* < 0.05). (c) Comparison of the grey values of P-P38 protein in renal cortex of rats (*n* = 3). The result showed that the grey value of P-P38 protein of DN group was significantly increased compared with NC group (*P* < 0.05); the grey value of P-P38 was significantly decreased in TXL group compared with DN group (*P* < 0.05). (d) Representative photograph of P38 staining (green) and cell nucleus (DAPI blue) in cultured podocyte. (e) Comparison of the fluorescence intensity of P38 protein in cultured podocyte. (f) Representative photograph of P-P38 staining (green) and cell nucleus (DAPI blue) in cultured podocyte. (g) Comparison of the fluorescence intensity of P-P38 protein in cultured podocyte. ^*∗*^ *P* < 0.05 versus NG. ^#^ *P* < 0.05 versus HG.

To explore the effect of TXL on P38 in podocyte, the expression of P38 and phosphorylated P38 was detected in our in vivo and in vitro studies. In our study, HG and TXL had no significant effect on total P38 expression in podocyte in vivo and in vitro (Figures 3(a), 3(b), and 3(d)). However, the expression of phosphorylated P38 was significantly increased in diabetic rats and high glucose cultured podocyte compared with NC group (Figures 3(a), 3(c), and 3(e)). Meanwhile, TXL significantly decreased the expression of phosphorylated P38 in diabetic rats and high glucose cultured podocyte (Figures 3(a), 3(c), and 3(e)).

### 3.4. TXL Regulated the Expression of Bcl-2, Bax, and Caspase-3 in Diabetic Rats and High Glucose Cultured Podocyte

**Figure 4:**
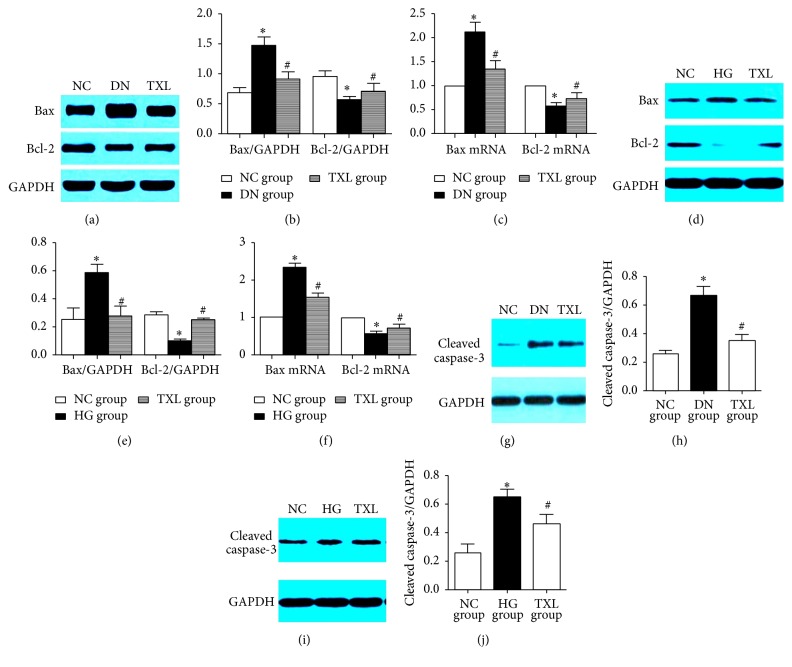
Effect of TXL on Bax, Bcl-2, and caspase-3 expression in vivo and in vitro. (a) Representative band of Bax and Bcl-2 protein in renal cortex of rats. (b) Comparison of the grey values of Bax and Bcl-2 protein in renal cortex of rats (*n* = 3). The result showed that the grey value of Bax protein was significantly increased and the grey value of Bcl-2 protein was significantly decreased in DN group compared with NC group (*P* < 0.05). The grey value of Bax protein was significantly decreased and the grey value of Bcl-2 protein was significantly increased in TXL group compared with DN group (*P* < 0.05). (c) Comparison of mRNA levels of Bax and Bcl-2 in renal cortex of rats (*n* = 3). The result showed that Bax mRNA expression was significantly increased and Bcl-2 mRNA expression was significantly decreased in DN group compared with NC group (*P* < 0.05); Bax mRNA expression was significantly decreased and Bcl-2 mRNA expression was significantly increased in TXL group compared with DN group (*P* < 0.05). (d) Representative band of Bax and Bcl-2 protein in cultured podocyte. (e) Comparison of the grey values of Bax and Bcl-2 protein in cultured podocyte (*n* = 3). The result showed that the grey value of Bax protein was significantly increased and the grey value of Bcl-2 protein was significantly decreased in HG group compared with NC group (*P* < 0.05). The grey value of Bax protein was significantly decreased and the grey value of Bcl-2 protein was significantly increased in TXL group compared with HG group (*P* < 0.05). (f) Comparison of mRNA levels of Bax and Bcl-2 in cultured podocyte (*n* = 3). The result showed that Bax mRNA expression was significantly increased and Bcl-2 mRNA expression was significantly decreased in HG group compared with NC group (*P* < 0.05); Bax mRNA expression was significantly decreased and Bcl-2 mRNA expression was significantly increased in TXL group compared with HG group (*P* < 0.05). (g) Representative band of cleaved caspase-3 protein in renal cortex of rats. (h) Comparison of the grey values of cleaved caspase-3 protein in renal cortex of rats (*n* = 3). The result showed that the grey value of cleaved caspase-3 was significantly increased in DN group compared with NC group (*P* < 0.05); the grey value of cleaved caspase-3 was significantly decreased in TXL group compared with DN group (*P* < 0.05). (i) Representative band of cleaved caspase-3 protein in cultured podocyte. (j) Comparison of the grey values of cleaved caspase-3 protein in cultured podocyte (*n* = 3). The result showed that the grey value of cleaved caspase-3 was significantly increased in HG group compared with NC group (*P* < 0.05); the grey value of cleaved caspase-3 was significantly decreased in TXL group compared with HG group (*P* < 0.05). ^*∗*^ *P* < 0.05 versus NG. ^#^ *P* < 0.05 versus HG.

In our in vivo and in vitro studies, the expression of Bax was significantly increased and the expression of Bcl-2 was significantly decreased in renal cortex of diabetic rats (Figures 4(a), 4(b), and 4(c)) and high glucose cultured podocyte (Figures 4(d), 4(e), and 4(f)). Administration of TXL resumed the balance of Bax and Bcl-2 expression. The expression of cleaved caspase-3 was then detected in our in vivo and in vitro studies. Cleaved caspase-3 expression was significantly increased in diabetic rats (Figures 4(g) and 4(h)) and high glucose cultured podocyte (Figures 4(i) and 4(j)) compared with NC group. Moreover, TXL significantly decreased cleaved caspase-3 expression in renal cortex of diabetic rats and high glucose cultured podocyte.

### 3.5. TXL Protected Podocyte from Apoptosis in Diabetic Rats and High Glucose Cultured Podocyte

**Figure 5:**
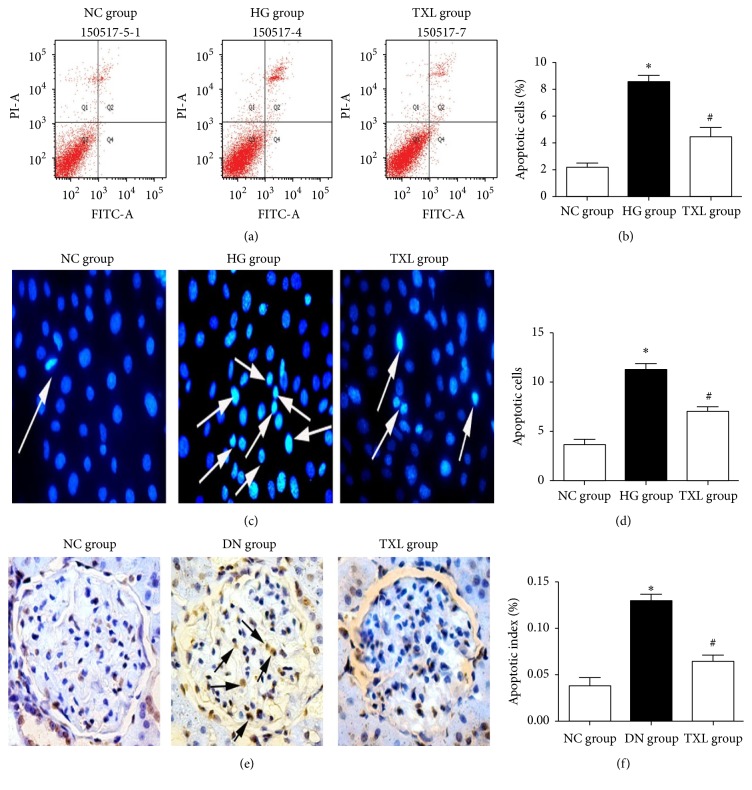
Effect of TXL on podocyte apoptosis in vivo and in vitro. (a) Representative photograph of flow cytometry analysis. (b) Comparison of apoptotic cells in cultured podocyte of different groups detected by flow cytometry. The result showed that the rate of apoptotic cells was significantly increased in HG group compared with NC group (*P* < 0.05); the rate of apoptotic cells was significantly decreased in TXL group compared with HG group (*P* < 0.05). (c) Representative photograph of Hoechst 33258 staining (arrows indicate apoptotic cells). (d) Comparison of apoptotic cells in cultured podocyte of different groups detected by Hoechst 33258 staining. The result showed that the number of apoptotic cells was significantly increased in HG group compared with NC group (*P* < 0.05); the number of apoptotic cells was significantly decreased in TXL group compared with HG group (*P* < 0.05). (e) Representative photograph of TUNEL staining (arrows indicate apoptotic cells). (f) Comparison of apoptotic cells in glomerulus of different groups detected by TUNEL. The result showed that the apoptotic index was significantly increased in DN group compared with NC group (*P* < 0.05); the apoptotic index was significantly decreased in TXL group compared with DN group (*P* < 0.05). ^*∗*^ *P* < 0.05 versus NG. ^#^ *P* < 0.05 versus HG.

Podocyte apoptosis was detected using different methods in our in vivo and in vitro studies. Podocyte apoptosis in diabetic rats, detected by TUNEL staining, was significantly increased compared with NC group. Administration of TXL significantly decreased podocyte apoptosis in diabetic rats (Figures 5(e) and 5(f)). Similarly, podocyte apoptosis in high glucose cultured podocyte, detected by Hoechst 33258 staining and HITC/PI staining, respectively, was significantly increased compared with NC group. TXL significantly decreased podocyte apoptosis in high glucose cultured podocyte (Figures 5(a)–5(d)).

## 4. Discussion

As we know, podocyte apoptosis is a main pathological mechanism of DN. Clinical studies have demonstrated that podocyte density is decreased [20] and decreased podocyte number is a top predictor of albuminuria in DN patients [21, 22]. Meanwhile, recent studies have found that podocyte apoptosis plays a key role in decreased podocyte number and progression of DN [23–26]. Thus, inhibition of podocyte apoptosis will be an important potential therapeutic target for DN. In accordance with previous studies, our study also found that podocyte apoptosis was significantly increased in diabetic rats and high glucose cultured podocyte. More importantly, TXL significantly alleviated podocyte apoptosis in our in vivo and in vitro studies.

Evidence suggests that oxidative stress plays a key role in the initiation and progression of DN [6–8]. It has been demonstrated that increased ROS production can induce characterized abnormalities of renal structure in DN such as glomerular and tubular hypertrophy, extracellular matrix accumulation, and thickening of glomerular or tubular basement membranes [27–29]. Meanwhile, oxidative stress has intensive relationship with podocyte apoptosis in DN. Previous studies have found that HG induces the production of ROS in podocyte and increased ROS production triggers podocyte apoptosis in DN [10, 18]. TXL has exhibited its antioxidant effect in the treatment of cardiovascular diseases and cerebrovascular diseases [15, 16]. However, the effect of TXL on oxidative stress in podocyte in DN remains unclear. Thus, the effect of TXL on ROS production in podocyte in DN was explored in our study. In our in vitro study, HG significantly increased ROS production in cultured podocyte, which has been demonstrated by previous studies [10, 18]. More importantly, TXL significantly decreased ROS production in high glucose cultured podocyte.

Although many ways are involved in increased ROS production, it has been demonstrated that NADPH oxidase is the major source of ROS in podocyte in DN. Moreover, inhibition of the activation of NADPH oxidase significantly decreased podocyte apoptosis and prevented progression of DN [10]. Among many members of NADPH oxidase family, Nox-4 is the key enzymes in ROS production and podocyte apoptosis induced by oxidative stress in DN [18, 19]. Herein, Nox-4 expressions were detected in our in vivo and in vitro studies. Consistent with previous studies [10, 19], our results showed that Nox-4 expression was significantly increased in renal cortex of diabetic rats and high glucose cultured podocyte. More importantly, TXL significantly decreased Nox-4 expression in our in vivo and in vitro studies.

Increased ROS production induces cell injury by activating different stress-sensitive pathways. ASK1, upstream kinase of P38, can be activated by ROS, followed by the activation of P38 pathway [12]. P38 MAPK pathway is an important proapoptotic signaling in podocyte [5, 11]. It has been demonstrated that the activation of P38 can regulate Bcl-2 and Bax expression and induce dysfunction of mitochondria [30]. The dysfunction of mitochondria subsequently releases apoptogenic proteins and activates caspase-3, which finally result in cell apoptosis. To explore molecular link between podocyte apoptosis and oxidative stress in DN, P38, Bcl-2, Bax, and caspase-3 were detected in our study. Our results showed that P38 and caspase-3 were activated in diabetic rats and hyperglycemia-induced podocyte. Our study also found that Bax expression was significantly increased and Bcl-2 expression was significantly decreased in podocyte in vivo and in vitro. More importantly, TXL significantly inhibited the activation of P38 and caspase-3 and resumed the balance of Bax and Bcl-2 expression.

## 5. Conclusion

In conclusion, we speculated that TXL might have pleiotropic effect on oxidative stress and P38 pathway both indirectly and directly in podocyte, which may be a main mechanism of protected effect on podocyte apoptosis in DN. However, the other therapeutic target of TXL on podocyte injury in DN remains unclear. Thus, further studies will be performed in our later research.
